# Viral pathogens in effluent samples collected from a swine slaughterhouse in Central Italy: a 1-year molecular surveillance

**DOI:** 10.3389/fmicb.2026.1957130

**Published:** 2026-10-29

**Authors:** Gianluigi Ferri, Giovanni Loris Alborali, Alberto Olivastri, Maria Antonietta Piccioni, Adriana Ianieri, Alberto Vergara

**Affiliations:** 1 Department of Veterinary Medicine, Post-Graduate Specialization School in Food Inspection “G. Tiecco”, University of Teramo, Teramo, Italy; 2 Istituto Zooprofilattico Sperimentale della Lombardia e dell’Emilia-Romagna (IZSLER) “Bruno Umbertini”, Brescia, Italy; 3 SIAOA Veterinary Public Service, Ascoli Piceno, Italy; 4 Department of Food and Drugs, University of Parma, Parma, Italy

**Keywords:** effluent samples, environmental health, public health, swine slaughterhouse, viral pathogens

## Abstract

**Introduction:**

Molecular screening for zoonotic and species-specific viral pathogens in environmental samples is essential for obtaining epidemiological data to support risk-based assessments in the food industry, particularly in settings such as slaughterhouses.

**Methods:**

This study screened 72 effluent specimens collected between January and December 2025 from two sites within a domestic pig slaughterhouse in Central Italy: the stocking yard (V1) and the wastewater treatment plant (WWTP; V2). The viruses investigated were astrovirus, hepatitis A virus, hepatitis E virus (HEV), norovirus (NoV), and rotavirus (RV). Viral detection was performed using real-time reverse transcription quantitative polymerase chain reaction (RT-qPCR) and nested reverse transcription polymerase chain reaction (RT-PCR) assays, followed by phylogenetic analysis.

**Results:**

The results showed that ribonucleic acid (RNA) from at least one of the target viruses was detected in 37.50% of the specimens, with HEV3-c detected in 26.39%, NoV-GII.3 in 12.50%, and NoV-GI.4 in 9.72%. Among the positive effluent samples, 62.96% were collected at V1 and 37.04% at V2. Samples from V1 exhibited higher viral loads, with HEV RNA levels reaching (105) genome equivalents per milliliter (GE/mL) and NoV-GI and NoV-GII RNA levels reaching (103) GE/mL, compared with (103) GE/mL for HEV RNA and (101) GE/mL for NoV-GI and NoV-GII RNA in samples from V2.

**Discussion:**

The detection of viral RNA sequences both before and after WWTP treatment highlights scientific and regulatory concerns regarding the potential dissemination of zoonotic and species-specific viruses into the environment. Regulatory gaps in the management and monitoring of these environmental matrices underscore the importance of multidisciplinary approaches to addressing this One Health issue.

## 1 Introduction

The demand for safe fresh meat and meat-derived products has increased, alongside consistently high consumption levels across all continents, particularly in Asia and Africa, where the majority of demographic growth occurs (Food and Agriculture Organization, 2025).

These socio-economic conditions have driven improvements in production systems involving farms, abattoirs, and food industries. Among these settings, slaughterhouses represent crucial points for monitoring microbiological agents, including zoonotic and species-specific pathogens, and generating epidemiological data relevant to animal, human, and environmental health (García-Díez et al., 2023). Indeed, viral zoonotic agents, such as hepatitis E virus (HEV) and norovirus (NoV), may persist in the environment, depending on their occurrence in target hosts and/or spillover animal species, including humans (Geng et al., 2019).

Many studies have used molecular screening approaches to investigate specific hosts, particularly in food-producing animals. More specifically, the *Suidae* family, which includes hosts of many zoonotic viruses, such as HEV, and ruminants, which may act as spillover hosts, have been investigated in epidemiological studies involving the collection of specific tissues (e.g., liver, bile, and muscle), sera, and fecal samples (Uema et al., 2022; Ianiro et al., 2024; Santos-Silva et al., 2024). Similar approaches have also been applied to veterinarians and slaughterhouse workers by testing for viral ribonucleic acid (RNA) and measuring corresponding immunoglobulin titers (Mrzljak et al., 2021; Alandijany et al., 2025), and to abattoir surfaces (Ianiro et al., 2024).

These findings have prompted further research into the roles of the environment and occupational exposure, while also considering the potential implications for by-products, such as effluents, and their management (Saravanakumar et al., 2022). In this context, a few studies have investigated slaughterhouse effluent samples for viral RNA, including that of astrovirus (AstV), hepatitis A virus (HAV), HEV, NoV, and rotavirus (RV). Specifically, dos Santos et al. (2011) in Brazil and Fenaux et al. (2018) in France investigated effluents exclusively for HEV RNA. Subsequent studies expanded the range of viral pathogens investigated to include additional zoonotic and species-specific viruses and placed greater emphasis on evaluating the efficacy of wastewater treatment plants (WWTPs) at domestic pig abattoirs (Barrios et al., 2018; Rusiñol et al., 2026).

Against this background, this study aimed to provide environmental data on the circulation of viral RNA sequences in effluent samples collected from two sites at a domestic pig slaughterhouse in Central Italy: the stocking yard and the wastewater treatment plant (WWTP). To our knowledge, this is the first Italian study to investigate in depth the role of abattoir wastewater as a potential vehicle for viruses. The study highlights the persistence of viral RNA after WWTP treatment and the absence of viral parameters from the microbiological parameters specified in European Directive No. 271/1991 (transposed into the Italian Law No. 152/2006, 2006). Therefore, this study could serve as a starting point for further investigations to inform future effluent management and help protect public health.

## 2 Materials and methods

### 2.1 Sample collections

Between January and December 2025, a total of 72 effluent samples were collected from a pig slaughterhouse located in the Marche region of Central Italy to investigate the presence of viral RNA sequences from the following pathogens in the effluent: AstV, HAV, HEV, NoV GI, NoV GII, and RV. During the study period, 8,322 domestic pigs (*Sus scrofa domesticus*) were slaughtered, corresponding to a reported mean of 232 slaughtered animals per day, with peaks of more than 400 animals per day during the winter season. During this season, two swine populations were represented: pigs from intensive farms and pigs raised for domestic self-consumption, the latter reflecting a longstanding tradition in Central Italy. Therefore, on each sampling day, the veterinary public health authorities provided data on slaughtering volumes (i.e., the number of pigs slaughtered per sampling day) and other relevant information, such as whether the pigs originated from intensive farms or domestic breeding operations. A detailed overview is provided in Table 1.

**Table 1:** A representation of domestic pigs’ slaughtering volumes in the year 2025.

Samples’ ID	Date	N. slaughtered pigs/day	IF	DF
W1	Jan_09_2025	232	227	5
W2	Jan_13_2025	179	159	20
W3	Jan_27_2025	283	275	8
W4	Feb_06_2025	292	288	4
W5	Feb_14_2025	303	300	3
W6	Feb_24_2025	269	268	1
W7	Mar_06_2025	229	229	–
W8	Mar_17_2025	157	157	–
W9	Mar_27_2025	245	245	–
W10	Apr_03_2025	317	317	–
W11	Apr_14_2025	166	166	–
W12	Apr_24_2025	142	142	–
W13	May_05_2025	201	201	–
W14	May_15_2025	178	178	–
W15	May_26_2025	197	197	–
W16	Jun_05_2025	138	138	–
W17	Jun_16_2025	205	205	–
W18	Jun_26_2025	122	122	–
W19	Jul_03_2025	91	91	–
W20	Jul_14_2025	83	83	–
W21	Jul_24_2025	88	88	–
W22	Aug_04_2025	74	74	–
W23	Aug_14_2025	65	65	–
W24	Aug_25_2025	63	63	–
W25	Sep_04_2025	81	81	–
W26	Sep_15_2025	107	107	–
W27	Sep_25_2025	264	264	–
W28	Oct_02_2025	302	302	–
W29	Oct_13_2025	329	329	–
W30	Oct_23_2025	301	301	–
W31	Nov_03_2025	451	423	28
W32	Nov_13_2025	407	391	16
W33	Nov_24_2025	483	475	8
W34	Dec_04_2025	396	365	31
W35	Dec_15_2025	403	388	15
W36	Dec_23_2025	479	436	43
Total amounts		8,322	8,140	182

W, sampling days (which include V_1_ and V_2_ specimens); IF, intensive farms; DF, domestic farms.

The sampling procedures were designed and implemented at 10-day intervals over 1 year, resulting in three sampling events per month, as illustrated in Table 1. Two 1 L effluent aliquots were collected: the first from the collection tank/stocking yard and the second directly from the WWTP after the depuration process. The WWTP had water flows of 8,000 L/day and 56,000 L/week. More specifically, V_1_ included effluents from different areas of the slaughterhouse, such as the *antemortem* lairage area, stunning, bleeding, and evisceration rooms, and the meat-cutting section. The effluent flows are schematically illustrated by the violet dashed lines and arrows in Figure 1.

**Figure 1:**
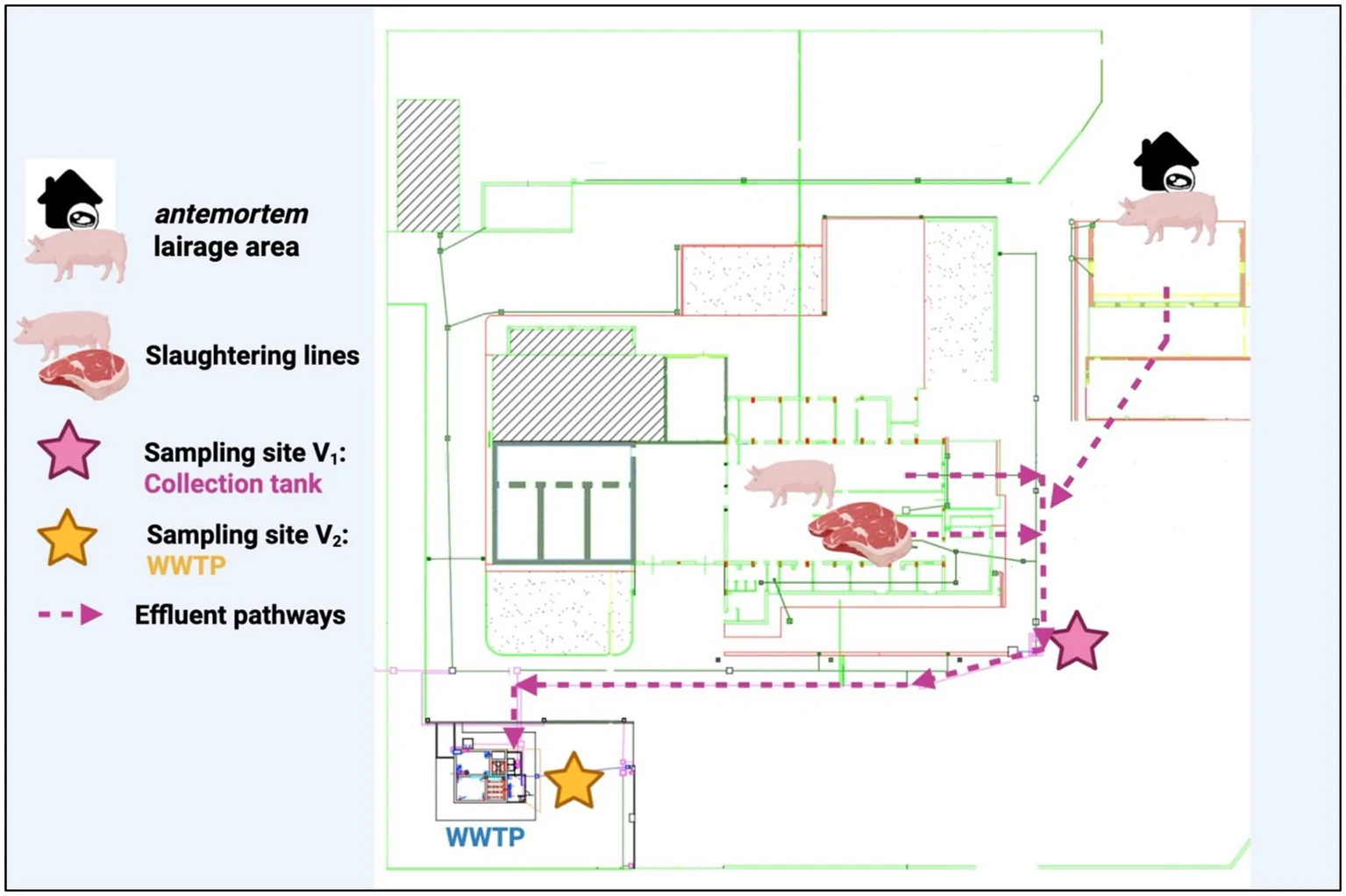
Schematic representation of the planimetry belonging to the involved swine slaughterhouse. Sample sites V_1_ and V_2_, and wastewater pathways from the collection tank to the wastewater treatment plant (WWTP) are indicated.

A total of 36 specimens were collected from the stocking yard (V_1_), and an equal number was collected after depuration at the WWTP (V_2_), as described similarly by dos Santos et al. (2011) (Figure 1).

Both samples were placed in sterile plastic bottles (VWR^®^, Water Sampling Bottles, Sterile, Milan, Italy) and transported to the laboratory under refrigerated conditions.

From a legal perspective, effluents from slaughterhouses are classified as industrial wastewater and require monitoring for specific parameters, including microbiological and chemical indicators (e.g., heavy metals and ammonia), in accordance with the European Directive No. 271/1991, as transposed into Italian Law No. 152/2006 (2006). These wastewaters are normally treated before being released into public sewage systems. More specifically, the WWTP investigated used a total oxidation activated-sludge biological system, as described in Annex 5, Table 3 of Italian Law No. 152/2006 (2006). This type of WWTP combines two treatment processes performed in separate tanks connected by pump systems: (1) metabolic processes carried out by aerobic bacteria in aerated tanks and (2) denitrification processes to reduce bacterial and chemical loads. The treatment processes were considered complete when the following chemical parameters reached values below the specific thresholds: total nitrogen ≤15 mg/L, total phosphorus ≤2 mg/L, and ammonia <15 mg/L, in accordance with Italian Law No. 152/2006 (2006). Effluent treatment concluded with chlorination. Other parameters were also required to be monitored, including an *Escherichia coli* count (ECC) < 5.0 × 10^3^ CFU/100 mL, total suspended solids <80 mg/L, and animal/vegetable oils and fats of <40 mg/L, among others (Italian Law No. 152/2006, 2006).

### 2.2 Sample processing and RNA extraction procedure

All V_1_ and V_2_ specimens underwent viral concentration procedures. Each 1.0-L sample was vortexed for 1 min, and five 15.0-mL aliquots were prepared in sterile Falcon tubes. The aliquots were then centrifuged at 3,000 × *g* for 10 min under refrigerated conditions. The resulting supernatants were subjected to two successive centrifugation steps at 180,000 × *g* for 2 h each. Finally, the supernatant was removed, the pellets were resuspended in 500 μL of RNase-free water (Invitrogen UltraPure DNase/RNase-Free Distilled Water, ThermoFisher™, Waltham, MA, United States), and the samples were stored at −80 °C until RNA extraction, in accordance with Nordgren et al. (2009) and dos Santos et al. (2011).

RNA was extracted from the obtained pellets using the TRIzol LS method (Invitrogen, Ltd., Paisley, United Kingdom). The protocol initially involved the addition of two reagents, TRIzol (250 μL/sample) and chloroform (100 μL/sample), at two separate steps to the initial sample volume of 500 μL. The subsequent steps involved two centrifugation procedures (12,000 × *g* for 15 min and 7,500 × *g* for 5 min), interspersed with alcoholic washes using 500 μL of ethanol and isopropyl alcohol, both at a concentration of 75.0%. The resulting pellets were resuspended in 50 μL of RNase-free water (Invitrogen UltraPure DNase/RNase-Free Distilled Water, ThermoFisher™, Waltham, MA, United States) and stored at −80 °C until the biomolecular assays were performed. Viral recovery and extraction efficiency were assessed in the V_1_ and V_2_ samples by adding a known concentration (10^5^ RNA copies) of a process control (Mengovirus vMC0) to each 15.0-mL aliquot before processing, as described in the international standard (ISO, 2017). Extraction efficiency was considered acceptable when recovery rates exceeded 1%. In addition, negative controls consisting of RNase-free water subjected to the same extraction protocol were included in each assay to prevent potential cross-contamination.

### 2.3 Biomolecular assays: real-time RT-qPCR and nested RT-PCR

All samples (RNA extracts from the concentrated pellets) were initially screened using one-step, real-time reverse transcription quantitative polymerase chain reaction (RT-qPCR) assays performed with the GENE UP^®^ System (bioMérieux, Paris, France). The resulting viral loads were expressed as genome equivalents per milliliter (GE/mL). Commercial pathogen-specific kits (ceeramTools; Thermo Fisher Scientific™, Waltham, MA, United States) specific to the investigated pathogens were used. Real-time RT-qPCR assays were performed in duplicate for each extracted RNA sample. PCR inhibition was systematically assessed by analyzing each sample as both undiluted RNA and a 1:10 dilution in RNase-free water, while also monitoring the internal amplification controls included in the commercial kits. Specifically, a sample was considered free of inhibition if the cycle threshold (C_t_) shift between the undiluted and diluted RNA samples was consistent with theoretical expectations (ΔC_t_ ≈ 3.32 ± 0.5) or if the C_t_ values remained within ± 1.0 C_t_ of the negative control. Experimental reliability was assessed using RNA-positive controls and no-template negative controls in each run. According to the manufacturer’s instructions, the final reaction volume was 25 μL, comprising 20 μL of reagents and 5 μL of RNA template. Positive and negative controls were also included (also provided by the kits used). The GE/mL were determined by comparing the observed amplification curves with standard curves generated using known concentrations ranging from 10^7^ to 10^1^ GE/mL. The 520-nm channel was used to detect fluorescence. The limits of detection (LOD) and quantification (LOQ) were determined by performing 10-fold dilutions starting from 10^7^ to 10^1^ GE per 2.5 mL of specimens. More specifically, the lowest concentration of MS2-PLP was defined as the LOD (characterized by a 100% probability of detection), whereas the LOQ represented the lowest concentration of a specific analyte that could be quantified, as described by Kralik and Ricchi (2017).

According to the manufacturer’s instructions, the thermocycling conditions were identical for all investigated pathogens (AstV, HAV, HEV, NoV-GI, NoV-GII, and RV). These conditions consisted of an initial reverse transcription step at 45 °C for 10 min, followed by denaturation at 95 °C for 10 min, and 40 cycles composed of two steps: 95 °C for 15 s and 60 °C for 45 s.

All positive profiles identified during quantitative molecular screenings were confirmed using nested reverse transcription polymerase chain reaction (RT-PCR) assays. The reactions were performed in final volumes of 25 μL, and the thermocycling conditions followed the respective published protocols for all investigated viruses: AstV (Noel et al., 1995), HAV (Taffon et al., 2011), HEV (Huang et al., 2002), NoV-GI and NoV-GII (Lew et al., 1994), and RV (Ben Salem et al., 2010).

The resulting PCR products were loaded onto agarose gels with concentrations ranging from 1.5 to 2.0%, depending on the amplicon sizes. Suspected positive amplicons were compared with 50-bp and 100-bp DNA ladders (FastGene^®^, Nippon Genetics, Düren, Germany). Before Sanger sequencing, the amplicons were purified using the QIAquick^®^ PCR Purification Kit (Qiagen, Hilden, Germany). The resulting sequences were inspected, and low-quality ends were trimmed using CHROMAS software (version 2.6.6; Technelysium Pty Ltd., Australia). Query sequences were compared with reference sequences from the NCBI GenBank database using the BLASTN tool (accessed 18 December 2025).^1^ Sequence alignment was performed using the CLUSTAL W algorithm integrated into MEGA 12 software. The final alignments included sequences of 320 bp for NoV-GI (ORF2/capsid region), 310 bp for NoV-GII (ORF2/capsid region), and 348 bp for HEV (ORF2 region). Reference sequences were selected according to high BLASTN similarity and the availability of representative prototype sequences for the assigned genogroups or genotypes in GenBank. Model selection was performed using MEGA 12, and the Kimura 2-parameter model coupled with a gamma distribution was used as the nucleotide substitution model.

The sequences were subsequently deposited in GenBank^2^ under their respective accession numbers (accessed on 23 and 27 January 2026). Phylogenetic trees were constructed using the neighbor-joining method in MEGA 12 software, and node robustness was evaluated using 1,000 bootstrap replicates. The identified genotypes and sub-lineages were assigned according to the clustering with reference strains of known classification. Bootstrap values were expressed as percentages (0–100%) in the figures and their corresponding legends. The trees were exported in Newick format and uploaded to the open-source Interactive Tree of Life (iTOL) software (Heidelberg, Germany) for tree construction. All discovered sequences were highlighted in light green in the respective phylogenetic trees constructed for each viral pathogen.

### 2.4 Bacteriological indicators

All V_1_ and V_2_ samples were also screened for quantitative microbiological indicators using standardized protocols. The parameters included the total bacterial count (TBC), determined using plate count agar (PCA; Oxoid Ltd., Basingstoke, Hampshire, United Kingdom) in accordance with ISO (1999), and the total coliform count (TCC) and ECC, determined in accordance with ISO (2012). Serial tenfold dilutions of the samples were prepared using Maximum Recovery Diluent (MRD–Oxoid Ltd., Basingstoke, Hampshire, United Kingdom). The results were expressed as colony-forming units per milliliter (CFU/mL).

### 2.5 Statistical analysis

All statistical analyses were conducted using IBM^®^ SPSS Statistics version 20.0 (SPSS, Chicago, IL, United States). The Shapiro–Wilk test was used to assess the normality of the viral load distributions, expressed in GE/mL. Samples with values below the LOD or LOQ were assigned a value of LOD/√2 for quantitative statistical analysis. Viral loads (GE/mL) between the two sampling sites (V_1_ and V_2_), sampled on the same day, were compared using a paired, two-tailed *t*-test. A two-way analysis of variance (ANOVA) was used to assess the effects of seasonality and daily slaughter volumes, considered independent variables, on viral loads (GE/mL), the dependent variable. For the statistical comparisons performed, the degrees of freedom (df), *p*-values, and effect sizes were reported. Effect sizes were expressed as Pearson’s correlation coefficient (*r*). The 95% confidence intervals (CIs) for all percentages were calculated using the Clopper–Pearson method. Statistical significance was set at *p* < 0.05.

## 3 Results

A total of 72 wastewater samples collected during the monitoring period (36 V_1_ and 36 V_2_ samples) were tested for viral RNA. Of these, 37.50% (27/72; 95% CI: 32.03–42.97%) were positive for at least one of the investigated viral pathogens, and viral RNA co-detections were observed in 11.11% (8/72; 95% CI: 3.85–18.37%) of the specimens. When samples with either single detections or co-detections were considered, the overall proportion of positive samples was 48.61% (35/72; 95% CI: 37.07–60.15%).

Among the individual pathogens detected, HEV RNA was the most frequently identified, with a prevalence of 26.39% (19/72; 95% CI: 16.21–36.57%) among all collected specimens. When calculated relative to the total number of positive viral detection profiles, HEV RNA accounted for 54.28% (19/35; 95% CI: 37.78–70.78%) of the detections.

Norovirus RNA was detected in 22.22% (16/72; 95% CI: 12.62–31.82%) of the V_1_ and V_2_ samples and accounted for 45.71% (16/35; 95% CI: 29.21–62.21%) of the positive detection profiles. Regarding norovirus genogroups, NoV-GII was detected in 12.50% (9/72; 95% CI: 4.86–20.14%) of all samples and accounted for 25.71% (9/35; 95% CI: 11.23–40.19%) of the positive profiles. NoV-GI was detected in 9.72% (7/72; 95% CI: 2.88–16.56%) of all samples and accounted for 20.00% (7/35; 95% CI: 6.75–33.25%) of the positive profiles.

AstV, HAV, and RV were not detected in either V_1_ or V_2_ specimens.

Regarding the distribution of positive samples by sampling site, 23.61% (17/72; 95% CI: 13.80–33.42%) of the V_1_ samples and 13.89% (10/72; 95% CI: 5.91–21.87%) of the V_2_ samples were positive for at least one of the investigated pathogens.

HEV RNA was detected in 27.78% (10/36; 95% CI: 13.15–42.41%) of V_1_ specimens and 25.00% (9/36; 95% CI: 10.86–39.14%) of V_2_ specimens. NoV-GII RNA was detected in 22.22% (8/36; 95% CI: 8.64–35.80%) of V_1_ specimens and 2.78% (1/36; 95% CI: 0.22–3.36%) of V_2_ specimens. Finally, NoV-GI RNA was detected in 11.11% (4/36; 95% CI: 0.85–21.37%) of V_1_ specimens and 8.33% (3/36; 95% CI: 0.31–16.35%) of V_2_ specimens.

In addition, positive viral detection profiles in V_1_ samples were reportedly also observed in their corresponding V_2_ samples collected on the same day (W), and no V_2_ specimens were positive exclusively. Among V_1_ specimens, 25.00% (9/36; 95% CI: 10.86–39.14%) were positive only at this sampling site and negative at the corresponding V_2_ site. Among these nine V1-only positive specimens, NoV-GII was detected in 77.78% (7/9; 95% CI: 50.62–100.0%), whereas NoV-GI and HEV were each detected in 11.11% (1/7; 95% CI: 1.33–20.89%).

A detailed and schematic representation is provided in Table 2.

**Table 2:** Distributions of positive samples among V_1_ and V_2_ collected ones.

Collection dates	Positive samples’ ID	Types (V_1_–V_2_)	Discovered viral pathogens
Jan_13_2025	W2	V_1_	HEV; NoV-GII
		V_2_	HEV
Jan_27_2025	W3	V_1_	NoV-GII
		V_2_	–
Feb_06_2025	W4	V_1_	NoV-GII
		V_2_	–
Feb_14_2025	W5	V_1_	NoV-GII
		V_2_	–
Feb_24_2025	W6	V_1_	NoV-GII
		V_2_	–
Mar_06_2025	W7	V_1_	NoV-GII
		V_2_	–
Mar_17_2025	W8	V_1_	HEV
		V_2_	HEV
Mar_27_2025	W9	V_1_	HEV
		V_2_	–
Apr_03_2025	W10	V_1_	NoV-GII
		V_2_	–
May_26_2025	W15	V_1_	HEV, NoV-GII
		V_2_	HEV, NoV-GII
Aug_25_2025	W24	V_1_	HEV
		V_2_	HEV
Sep_04_2025	W25	V_1_	HEV
		V_2_	HEV
Sep_15_2025	W26	V_1_	HEV, NoV-GI
		V_2_	HEV, NoV-GI
Oct_02_2025	W28	V_1_	NoV-GI
		V_2_	NoV-GI
Nov_24_2025	W33	V_1_	HEV, NoV-GI
		V_2_	HEV, NoV-GI
Dec_04_2025	W34	V_1_	HEV
		V_2_	HEV
Dec_15_2025	W35	V_1_	HEV, NoV-GI
		V_2_	HEV

W, sampling days (which include V_1_ and V_2_ specimens); HEV, hepatitis E virus; NoV-GI and NoV-GII, norovirus genogroups I and II.

The mean viral load was 10^2^ GE/mL, while the highest viral load was 10^5^ GE/mL, detected in two specimens positive for HEV RNA. Viral loads of 10^3^ GE/mL were observed for both NoV genogroups I and II. The Shapiro–Wilk test indicated no significant departure from normality in the viral load data (*W* = 0.962, *p* = 0.184). The paired, two-tailed *t*-test revealed a statistically significant difference between the paired V_1_ and V_2_ samples (df = 35, *p* = 0.041, effect size *r* = 0.397). When each pathogen was considered separately, HEV RNA detection showed a statistically significant difference between V_1_ and V_2_ specimens (df = 10, *p* = 0.040, and effect size *r* = 0.586). No statistically significant differences were observed for NoV-GI (df = 3, *p* = 0.804, and effect size *r* = 0.500) or NoV-GII (df = 7, *p* = 0.158, and effect size *r* = 0.382).

Considering the independent variables (seasonality and daily slaughtered volumes) and viral loads (GE/mL), the two-way analysis of variance (ANOVA) model showed statistically significant differences (df = 18, *p* = 0.043, and effect size *r* = 0.81) between cold months (W1-W8 and W28-W36), which were characterized by high peaks of slaughtered pigs (483 animals/day), and higher viral RNA loads (HEV—10^5^ GE/mL and NoV-GI and NoV-GII—10^3^ GE/mL), compared with the spring–summer ones (from W9 to W27 HEV—10^3^ GE/mL and NoV-GI and NoV-GII—10^1^ GE/mL). A detailed overview of positive samples by collection month and the corresponding slaughtering volumes is provided in Table 3.

**Table 3:** Discovered viral loads from V1 and V2 positive samples and slaughtered pigs distributed among the year.

Pathogens	Positive samples’ ID	Types (V_1_-V_2_)	Viral loads (GE/mL)	Months	Slaughtered pigs/day
HEV	W2	V_1_ V_2_	10^3^ GE/mL 10^3^ GE/mL	January	179 (I-159; D-20)
	W8	V_1_ V_2_	10^5^ GE/mL 10^3^ GE/mL	March	157 (I-137; D-0)
	W9	V_1_	10^1^ GE/mL		245 (I-245; D-0)
	W15	V_1_ V_2_	10^3^ GE/mL 10^2^ GE/mL	May	197 (I-197; D-0)
	W24	V_1_ V_2_	10^3^ GE/mL 10^3^ GE/mL	August	63 (I-63; D-0)
	W25	V_1_ V_2_	10^3^ GE/mL 10^3^ GE/mL	September	81 (I-81; D-0)
	W26	V_1_ V_2_	10^1^ GE/mL 10^1^ GE/mL		107 (I-107; D-0)
	W33	V_1_ V_2_	10^2^ GE/mL 10^2^ GE/mL	November	483 (I-475; D-8)
	W34	V_1_ V_2_	10^5^ GE/mL 10^3^ GE/mL	December	396 (I-365; D-31)
	W35	V_1_ V_2_	10^5^ GE/mL 10^2^ GE/mL		403 (I-388; D-15)
NoV-GI	W26	V_1_ V_2_	10^3^ GE/mL 10^3^ GE/mL	September	107 (I-107; D-0)
	W28	V_1_ V_2_	10^1^ GE/mL 10^1^ GE/mL	October	302 (I-302; D-0)
	W33	V_1_ V_2_	10^3^ GE/mL 10^3^ GE/mL	November	483 (I-475; D-8)
	W35	V_1_	10^1^ GE/mL	December	403 (I-388; D-15)
NoV-GII	W2	V_1_	10^1^ GE/mL	January	179 (I-159; D-20)
	W3	V_1_	10^1^ GE/mL		283 (I-275; D-8)
	W4	V_1_	10^1^ GE/mL	February	292 (I-288; D-4)
	W5	V_1_	10^1^ GE/mL		303 (I-300; D-3)
	W6	V_1_	10^1^ GE/mL		269 (I-268; D-1)
	W7	V_1_	10^1^ GE/mL	March	229 (I229; D-0)
	W10	V_1_	10^1^ GE/mL	April	317 (I-317; D-0)
	W15	V_1_ V_2_	10^2^ GE/mL 10^1^ GE/mL	May	197 (I-197; D-0)

I, intensive farms; D, domestic farms for self-consumption; HEV, hepatitis E virus; NoV-GI and NoV-GII, norovirus genogroups I and II.

All samples positive by RT-qPCR (19 for HEV, 7 for NoV-GI, and 9 for NoV-GII) were confirmed using nested RT-PCR, and the corresponding amplicons were subjected to Sanger sequencing, as described in the Materials and Methods section. For HEV, high-quality sequences were successfully obtained from all 19 positive samples. Comparative sequence alignment revealed genetic variability among the amplicons, resulting in six representative sequences deposited in GenBank (PX925942, PX925944, PX925945, PX925946, PX925947, and PX925948). For NoV-GI, two positive samples yielded sequences identical to the deposited variant PX910003, whereas the remaining five samples yielded two additional deposited sequences (PX910041 and PX909882). For NoV-GII, sequences obtained from nine positive samples were grouped into five representative sequences (PX924506, PX924507, PX924408, PX924412, and PX924417).

The HEV genetic sequences identified in this study comprised fragments of the ORF2 region. BLASTN analysis revealed 100% nucleotide identity with HEV genotype 3 subtype 3c. The sequences were deposited in GenBank under the following accession numbers: PX925942, PX925944, PX925945, PX925946, PX925947, and PX925948. Their sequence similarity profiles (100.0% nucleotide identity) included matches to HEV sequences obtained from human blood and/or serum, domestic pig feces, and sewage effluents, as illustrated in Figure 2, which presents the corresponding phylogenetic analysis.

**Figure 2:**
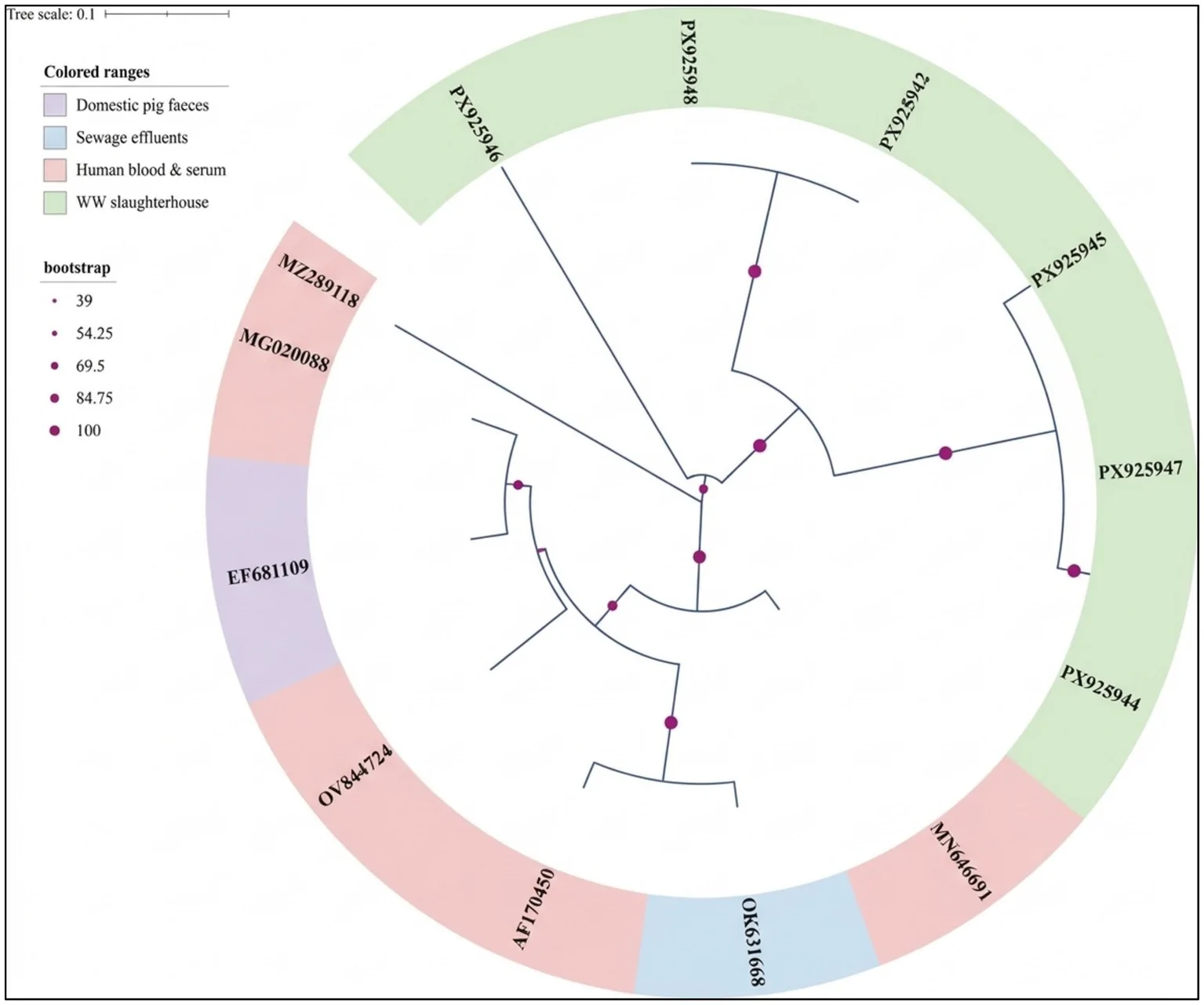
Phylogenetic relationship between the hepatitis E virus (HEV) RNA sequences detected in wastewater collected from a pig slaughterhouse in Central Italy and those of reference isolates, inferred using the neighbor-joining method (Kumar et al., 2018). Bootstrap support values (1,000 replicates) are expressed as percentages (%). Sequences obtained in this study are highlighted in light green.

As shown in Figure 2, all registered sequences showed 100.0% nucleotide identity with MN646691, MG02008, OV844724, and MZ289118, which are highlighted in red in the phylogenetic tree. These sequences were identified in blood and serum samples collected from human patients in the European continent (France, Germany, Switzerland, and Spain, respectively); all of them were HEV genotype 3. This high percentage of similarity was also observed for the following accession numbers: EF681109 (violet in the previous phylogenetic tree) and OK631668 (light blue). EF681109 was identified by Caprioli et al. (2007) in fecal samples from healthy pigs aged 2–5 months raised in north-central Italy. OK631668 was amplified from effluent water samples collected at a WWTP in Pakistan.

BLASTN analysis identified the positive amplicons from this study as NoV-GI.4. The corresponding GenBank accession numbers were PX910003, PX910041, and PX909882 (Figure 3).

**Figure 3:**
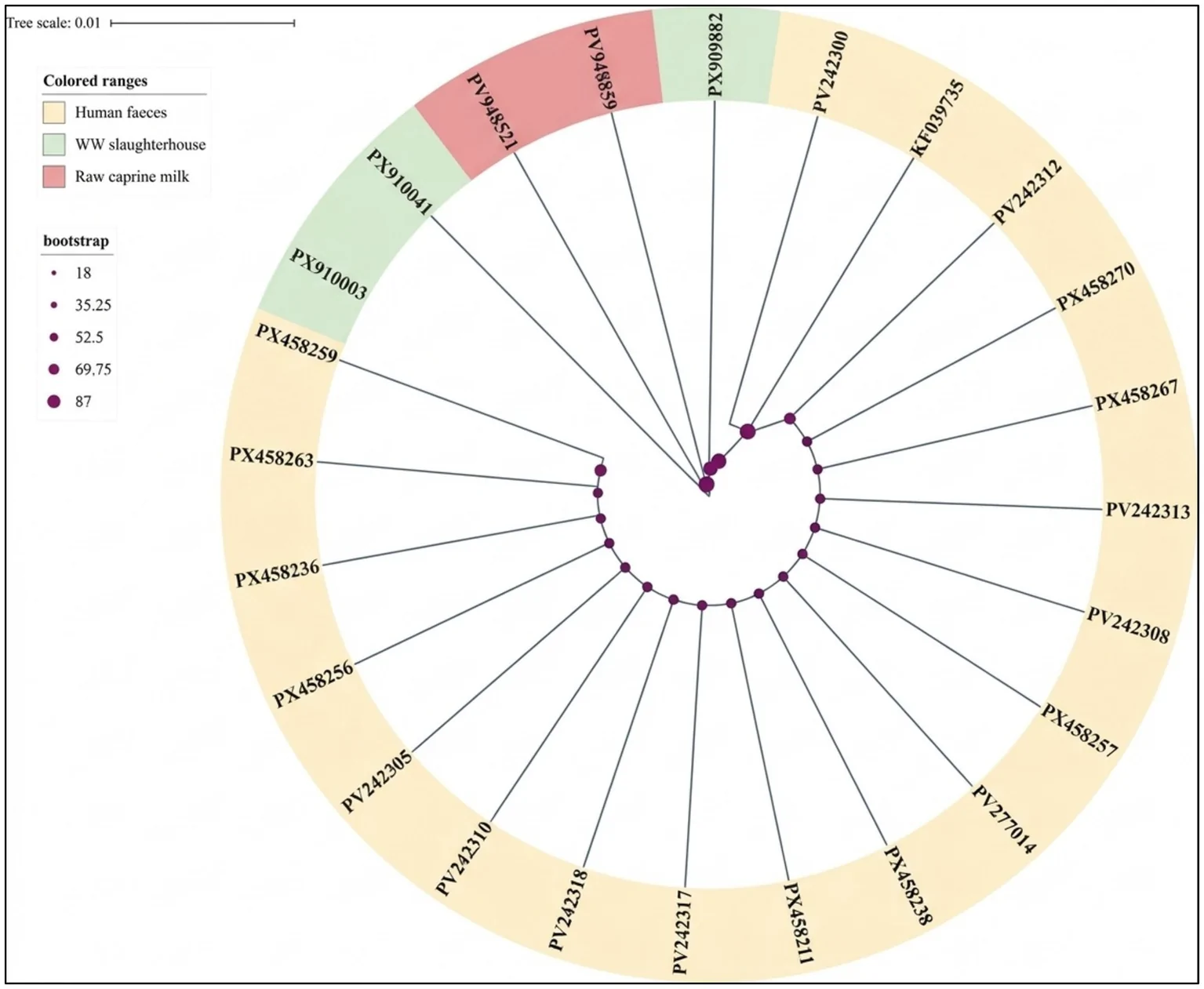
Phylogenetic relationships between the norovirus genogroup I.4 (NoV-GI.4) RNA sequences detected in wastewater collected from a pig slaughterhouse in Central Italy and those of reference isolates, inferred using the neighbor-joining method (Kumar et al., 2018). Bootstrap support values (1,000 replicates) are expressed as percentages (%). Sequences obtained in this study are highlighted in light green.

All sequenced samples showed 100.0% nucleotide identity with PV948521 and PV948859, highlighted in red in the phylogenetic tree. These NoV-GI.4 sequences were identified in unpasteurized goat milk collected from healthy goats in Abruzzo region of Central Italy. The sequences highlighted in yellow in Figure 3 showed an average nucleotide identity of 99.08% with NoV-GI.1 sequences identified in human fecal samples from the United States of America (USA).

Concerning NoV-GII, the obtained purified sequences, were analyzed and registered with these accession numbers: PX924506, PX924507, PX924408, PX924412, PX924417 and classified as NoV-GII.3. The highest similarities (nt. ID 100.0%) were observed for OP279621 and OP279623 (Figure 4) sequences that were discovered from sewage effluent samples collected from a WWTP located in Lombardia region (Northern Italy) and in South Korea ones (MW532592, MW532688, MW532638, MW532685, MW532613, MZ027014, and PP587395).

**Figure 4:**
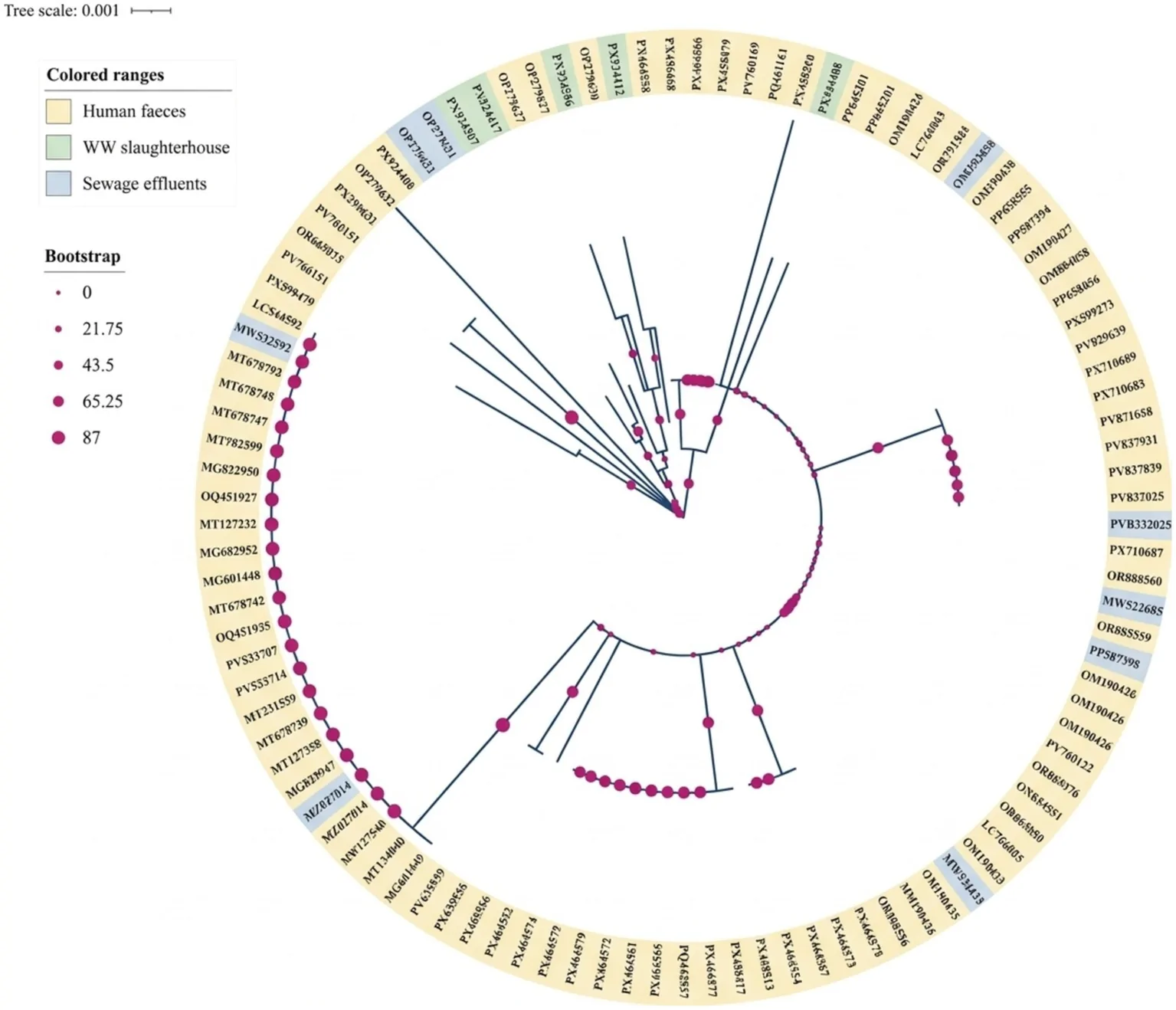
Phylogenetic relationships between the norovirus genogroup II.3 (NoV-GII.3) RNA sequences detected in wastewater collected from a pig slaughterhouse in Central Italy and those of reference isolates, inferred using the neighbor-joining method (Kumar et al., 2018). Bootstrap support values (1,000 replicates) are expressed as percentages (%). Sequences obtained in this study are highlighted in light green.

The quantitative microbiological analyses revealed high bacterial loads in both V_1_ and V_2_ samples. Among V_1_ samples, TBC values ranged from 1.37 × 10^9^ CFU/mL to 1.82 × 10^9^ CFU/mL, with a mean of 1.59 × 10^9^ CFU/mL. TCC values ranged from 6.81 × 10^7^ CFU/mL to 8.91 × 10^7^ CFU/mL, with a mean of 7.87 × 10^7^ CFU/mL. ECC values ranged from 3.34 × 10^6^ CFU/mL to 4.35 × 10^6^ CFU/mL, with a mean of 3.84 × 10^6^ CFU/mL. In V_2_ samples, TBC values ranged from 1.85 × 10^3^ CFU/mL to 2.38 × 10^3^ CFU/mL, with a mean of 2.10 × 10^3^ CFU/mL. TCC values ranged from 1.12 × 10^2^ CFU/mL to 1.46 × 10^2^ CFU/mL, with a mean of 1.29 × 10^2^ CFU/mL. ECC values were below the detection limits.

## 4 Discussion

The present molecular study was designed as an environmental investigation focusing on the dissemination of zoonotic pathogens, including HEV, NoV-GI, and NoV-GII, through effluent samples collected from a domestic pig slaughterhouse in Central Italy. This rationale was based on the concept that the slaughterhouse environment can serve as a meeting point or crossroads for numerous microbiological agents, including zoonotic viruses (Hdaifeh et al., 2020). Although previous studies have primarily focused on Category 3 animal by-products, in accordance with EU Regulations: No. 1069/2009 and 142/2011, such as blood, feces, and liver samples collected directly from slaughter lines, sewage specimens have received comparatively little attention (Cê et al., 2023; López-Martínez et al., 2023; Ragasri and Sabumon, 2023). Indeed, the potential role of slaughterhouses effluents as pathways for viral dissemination or as environmental indicators of zoonotic viruses has been insufficiently characterized, particularly regarding their ecological, epidemiological, public health implications for humans, animals and the environment (dos Santos et al., 2011; Takuissu et al., 2022; Rusiñol et al., 2026). Therefore, this this study represents the first Italian investigation to use molecular methods to screen effluent samples collected at two sites: stocking yard (V_1_) and the WWTP after the depuration process (V_2_), which was reportedly performed in accordance with the European Directive No. 271/1991, as illustrated in Figure 1.

Using this approach, viral RNA sequences from zoonotic pathogens were detected in the 72 effluent samples collected from V_1_ and V_2_. The observed general trend, about positive specimens, permitted to find RNA amplicons from 37.50% (27/72; 95% CI: 32.03–42.97%) of effluents that were positive to at least of one of the considered viral pathogens, e.g., HEV, NoV-GI, and NoV-GII. Few studies have specifically investigated slaughterhouse effluents for these viruses. In Italy, to our knowledge, slaughterhouse effluents had not previously been screened for zoonotic viral pathogens. Therefore, the results were initially compared with studies that detected viral RNA from HEV, NoV-GI, and NoV-GII in WWTPs, sewage, rivers, and seawaters. In 2016, Iaconelli et al. (2017) conducted a one-year surveillance study of human enteric viruses in raw and treated sewage collected from six municipal WWTPs in the Piedmont region of northern Italy. They reported a prevalence of 57.14% among positive samples for norovirus, which was higher than the 37.50% reported in this study. This difference may reflect the distinct study settings: the present study focused specifically on slaughterhouse effluents, whereas Iaconelli et al. (2017) investigated wastewater from densely populated urban and industrial areas. La Rosa et al. (2007) also amplified genetic regions, belonging to the NoV-GI and GII, reporting a prevalence of 31.70% in sewage samples collected from a WWTP in the Lazio region of Central Italy. This result was broadly comparable to the norovirus findings of the present study. Conversely, La Rosa et al. (2017) detected HEV RNA exclusively in slurry samples collected from 10 pig farms in the Emilia-Romagna and Lombardia regions in northern-Italy, reporting a prevalence of 75.0%. Their study did not include effluent samples collected from slaughterhouses.

Internationally, Rusiñol et al. (2026) investigated multiple viruses, including AstV, HEV, NoV, and RV, in slurry and wastewater samples collected during annual surveillance at two swine slaughterhouses in Barcelona, Spain. Their molecular results showed the following prevalences: 8.9% NoV RNA, 0.4% HEV, and 1.75% RV resulting different to those ones described in this study, that were: 26.39% (19/72; 95% CI: 16.21–36.57%) HEV RNA, 22.22% (16/72; 95% CI: 12.62–31.82%) NoV, and RV was never amplified. Although differences were observed in the data, the findings highlight the critical importance of adopting a broad ecological perspective that integrates animal, human, and environmental health. This approach offers an innovative framework for monitoring viral behavior in microbiological and epidemiological contexts, such as slaughterhouses.

Starting from this article, the analysis of the scientific literature on this specific topic revealed that the majority of studies focused on a single pathogen. For example, dos Santos et al. (2011) focused on HEV RNA detection in a domestic pig slaughterhouse in Brazil, screening two effluent sample types, collected from two sites: the stocking yard (such as the V_1_ samples of this study) and the final waste site (V_2_). HEV RNA was detected in 50.0% of the six specimens examined. In comparison, the prevalence calculated in the present study was 26.39% (19/72; 95% CI: 16.21–36.57%). This difference may be attributable to the different sampling times adopted by the authors. This study performed a one-year surveillance trying to provide epidemiological data, and it was initially inspired by the approach provided by dos Santos et al. (2011) who, on the contrary, provided a picture, belonging to a specific moment, exclusively focused on the HEV RNA circulation from Brazilian slaughterhouses. In both studies, HEV genotype 3 was identified. More specifically, subtype 3c was detected in the present study, whereas subtype 3b was reported by dos Santos et al. (2011). HEV-3 is widely distributed among members of the family *Suidae*, which serve as important animal reservoirs and may contribute to viral transmission to other susceptible species, including humans. Although HEV infection in swine is generally asymptomatic, HEV-3 is responsible for zoonotic infections in humans and can occasionally lead to hospitalization (Zahmanova et al., 2023). Finally, the HEV RNA sequences deposited in GenBank were assigned the accession numbers PX925942, PX925944, PX925945, PX925946, PX925947, and PX925948 (Figure 2). These sequences exhibited high nucleotide identity (100.0%) with HEV sequences detected in both animal and human-derived samples. Specifically, the corresponding sequences included MN646691, MG02008, OV844724, and MZ289118, which were detected in human blood and serum samples from Europe; EF681109, identified in fecal samples from pigs raised in north-central Italy and described by Caprioli et al. (2007); and OK631668, detected in sewage samples collected from WWTP in Pakistan. These genetic findings reinforce the notion that HEV exhibits considerable environmental persistence, which may be influenced by the ultrastructural characteristics of its quasi-enveloped virions (Ahmad et al., 2022). This observation also highlights the potential role of different ecological niches as sources and routes of HEV transmission among susceptible hosts, with important implications for viral epidemiology.

Regarding NoV, 22.22% of the samples tested positive (16/72; 95% CI: 12.62–31.82%). To our knowledge, this represents the first report from Italy of norovirus genogroups I and II detected in effluent samples collected from a domestic pig abattoir. Barrios et al. (2018) screened 52 wastewaters samples collected from stocking yards and WWTPs at four Argentinian abattoirs, each representing a different animal species (swine, bovine, chicken, and equine), but did not detect NoV RNA. In contrast, Rusiñol et al. (2026) detected NoV RNA in 8.92% of effluent samples collected from stocking yards. These detections belonged to genogroup II, which was associated with porcine-specific NoV, differing from the human-associated NoV-GI and NoV-GII detected in the present study, with prevalence values of 9.72% (7/72; 95% CI: 2.88–16.56%) and 20.00% (7/35; 95% CI: 6.75–33.25%), respectively.

Another important aspect of the norovirus findings is the registration of eight sequence accession numbers in GenBank. As reported in the Results section, the sequences PX910003, PX910041, and PX909882 were classified as NoV-GI.4 and exhibited high nucleotide identity (100.0%) with sequences detected in human feces samples from Europe and North America, as well as in caprine raw milk from the Abruzzo region of central Italy. These relationships are highlighted in yellow in the phylogenetic tree presented in Figure 3.

For genogroup II, five sequences (PX924506, PX924507, PX924408, PX924412, PX924417) were deposited in GenBank. The phylogenetic analysis (Figure 4) revealed high nucleotide identity with reference sequences associated with human sources. More specifically, the highest nucleotide similarities were observed for sequences discovered from sewage collected from WWTPs located in Lombardia region (northern Italy) with the accession numbers OP279621 and OP279623 (light blue highlighted in the phylogenetic tree; Figure 4) and sequences: MW532592, MW532688, MW532638, MW532685, MW532613, MZ027014, and PP587395 discovered from feces and WWTPs in South Korea (yellow marked accession numbers; Figure 4). Although these genetic similarities indicate close evolutionary relationships with human-associated lineages, further epidemiological investigations are needed to conclusively determine the source of contamination. According to the scientific literature, NoV genogroups GI and GII, have been detected primarily in wastewater from WWTPs treating sewage originating from industries and urban areas (Huang et al., 2022; Mroweh et al., 2025). These findings highlight the environmental persistence of NoV, which may be associated with its structural characteristics and its ability to remain stable under extreme pH conditions and at both low and high temperatures (Winder et al., 2022).

Conversely, slaughterhouse effluents can be considered complex matrices that may serve as molecular sentinels within a One Health framework for the surveillance and monitoring of zoonotic and species-specific viral genomes. Their assessment could therefore provide additional insights into viral persistence in food-processing environments (Rusiñol et al., 2026).

Another important aspect to consider is the variation in viral loads (GE/mL) between V_1_ and V_2_ samples in relation to seasonal variability and the corresponding slaughtering volumes, expressed as the number of domestic pigs slaughtered on each sampling day. According to the statistical analyses, viral loads decreased significantly between V_1_ samples and their corresponding V_2_ samples, particularly for HEV RNA. One possible explanation for these reductions is the substantial initial dilution occurring in the stocking yard, where large volumes of effluent from different areas of the slaughterhouse are collected, including the *antemortem* lairage area, stunning, bleeding and evisceration rooms, and the first meat-cutting section. In addition, the final chlorination process may contribute to capsid disruption and/or RNA degradation. However, it is important to emphasize that the detection of viral RNA by RT-qPCR does not establish the integrity or infectivity of viral particles. Therefore, conclusions regarding virucidal effects or loss of infectivity require appropriate viability assays. Nevertheless, the findings support the hypothesis that dilution within the slaughterhouse stocking-yard system may contribute to reductions in detectable viral loads. A similar phenomenon was reported by Barrios et al. (2018), who described reductions on the order to 10^2^ copies/mL in effluents from Argentine abattoirs. In the present study, 25.0% of the V1 samples (9/36; 95% CI: 10.86–39.14%) tested positive at the stocking-yard stage but were negative at the V_2_ stage.

Seasonal variability was observed in both viral detection rates and viral loads, with significantly higher viral loads recorded during the winter months than during the warmer months. As summarized in Table 3, HEV reached 10^5^ GE/mL during winter, compared with 10^3^ GE/mL during the warmer months, while NoV-GI and NoV-GII reached 10^3^ GE/mL during winter and 10^1^ GE/mL during the warmer months. These findings indicate higher viral loads during colder periods, particularly autumn and winter. However, interpreting seasonal patterns in slaughterhouse effluents requires consideration of multiple environmental factors and operational dynamics, as seasonal decreases in temperature may coincide with peaks in the number of animals slaughtered. Low temperatures may also favor viral persistence in the environment by reducing temperature-related capsid damage and enzymatic degradation, potentially contributing to higher viral loads during winter. Therefore, seasonal peaks in slaughtering volumes may act in combination with lower temperatures to increase viral loads in effluent samples. A similar seasonal trend was reported by Rusiñol et al. (2026), who screened effluent samples collected from two sites, as in the present study (V_1_ and V_2_). They primarily detected RV, HEV, and NoV, with average viral loads ranging from 10^2^ to 10^3^ copies/mL during winter, compared with 10^1^ to 10^2^ copies/mL during summer. Unlike Rusiñol et al. (2026), the present study also considered daily slaughtering volumes. Winter therefore represents a particularly relevant period for epidemiological surveillance, as the abattoir processed larger numbers of animals during these months, potentially increasing the likelihood of viral circulation in effluents.

Bacteriological fecal contamination and bacterial counts in the collected effluent samples from both V_1_ and V_2_ also warrant consideration, as they provide additional information about the sanitary characteristics of the slaughterhouse. The parameters assessed were TBC, TCC, and ECC. The average values in V_1_ samples were 1.59 × 10^9^ CFU/mL for TBC, 7.87 × 10^7^ CFU/mL for TCC, and 3.84 × 10^6^ CFU/mL for ECC. In V_2_ samples, the corresponding values were 2.10 × 10^3^ CFU/mL for TBC and 1.29 × 10^2^ CFU/mL for TCC, while ECC levels were below the detection limit. According to these findings, the V_2_ samples complied with the ECC limit of 5.0 × 10^3^ CFU/100 mL specified in Italian Law No. 152/2006 (2006), which transposes European Directive No. 271/1991. These results support the effectiveness of the WWTP in reducing bacterial contamination. Nevertheless, the present study also highlights the need for greater attention to the remaining sanitary, environmental, and regulatory challenges associated with viral contamination. The current regulatory framework for WWTPs at slaughterhouses, as established under EU Directive No. 271/1991, has limitations regarding the management of environmental disseminations of both zoonotic and species-specific viruses. In addition to this regulatory gap, these viruses may exhibit considerable resistance to challenging environmental conditions, including low pH, low temperatures, and chlorination, potentially enabling them to persist within particular geographical area where suitable primary or spillover hosts are available (Winder et al., 2022). These findings underscore the importance of implementing molecular surveillance programs to monitor viral circulation across ecosystems, from wildlife environments to food-processing facilities, and to address the associated environmental and public health concerns.

## 5 Conclusion

This study represents the first investigation in Italy to focus the molecular screening of viral pathogens in effluents collected from a swine slaughterhouse. It aimed to provide an epidemiological overview of viral RNA circulation along the wastewater flow by examining two key sampling points: the stocking yard (V_1_) and the WWTPs (V_2_).

From a regulatory perspective, wastewater management at slaughterhouses is classified as industrial wastewater management under EU Directive 91/271/EEC. Among the parameters addressed by the current regulatory framework, microbiological indicators primarily focus on bacteria. Furthermore, the revised EU regulatory framework, under Directive (EU) 2024/3019, which is scheduled to replace the current directive from August 2027 introduces important challenges and priorities, including antimicrobial resistance, microplastics, and the promotion of a circular economy. However, viral pathogens are not explicitly addressed in this regulatory framework, highlighting a continuing gap in the management and monitoring of viral contamination in wastewater.

The inclusion of virus-specific parameters in wastewater monitoring is therefore necessary, given the widespread environmental dissemination of viral agents and their relevance to One Health. This concern is further reinforced by the potential persistence of viruses under adverse environmental and physicochemical conditions, which may reflect the structural stability of viral capsids within effluent matrices. In this context, molecular surveillance represents a valuable approach for characterizing viral RNA circulation and generating virological and epidemiological data on specific environments. Such surveillance could help identify potential routes of environmental dissemination and support the development of more comprehensive monitoring strategies.

## Data Availability

The datasets presented in this study can be found in online repositories. The names of the repository/repositories and accession number(s) can be found in the article/supplementary material.

## Footnotes

1
https://blast.ncbi.nlm.nih.gov/Blast.cgi?BLAST_SPEC=blast2seq&LINK_LOC=align2seq&PAGE_TYPE=BlastSearch

2
https://www.ncbi.nlm.nih.gov/genbank/
